# Validation and reliability of a modified sphygmomanometer for the assessment of handgrip strength in Parkinson´s disease

**DOI:** 10.1590/bjpt-rbf.2014.0081

**Published:** 2015-04-27

**Authors:** Soraia M. Silva, Fernanda I. Corrêa, Paula F. C. Silva, Daniela F. T. Silva, Paulo R. G. Lucareli, João C. F. Corrêa

**Affiliations:** 1 Programa de Pós-graduação em Ciências da Reabilitação, Universidade Nove de Julho (UNINOVE), São Paulo, SP, Brazil; 2 Programa de Pós-graduação em Biofotônica Aplicada às Ciências da Saúde, UNINOVE, São Paulo, SP, Brazil

**Keywords:** Parkinson's disease, muscle strength dynamometer, reproducibility of results, aged

## Abstract

**BACKGROUND:**

Handgrip strength is currently considered a predictor of overall muscle strength and functional capacity. Therefore, it is important to find reliable and affordable instruments for this analysis, such as the modified sphygmomanometer test (MST).

**OBJECTIVES:**

To assess the concurrent criterion validity of the MST, to compare the MST with the Jamar dynamometer, and to analyze the reproducibility (i.e. reliability and agreement) of the MST in individuals with Parkinson's disease (PD).

**METHOD:**

The authors recruited 50 subjects, 24 with PD (65.5±6.2 years of age) and 26 healthy elderly subjects (63.4±7.2 years of age). The handgrip strength was measured using the Jamar dynamometer and modified sphygmomanometer. The concurrent criterion validity was analyzed using Pearson's correlation coefficient and a simple linear regression test. The reproducibility of the MST was evaluated with the coefficient of intra-class correlation (ICC_2,1_), the standard error of measurement (SEM), the minimal detectable change (MDC), and the Bland-Altman plot. For all of the analyses, α≤0.05 was considered a risk.

**RESULTS:**

There was a significant correlation of moderate magnitude (r≥0.45) between the MST and the Jamar dynamometer. The MST had excellent reliability (ICC_2,1_≥0.7). The SEM and the MDC were adequate; however, the Bland-Altman plot indicated an unsatisfactory interrater agreement.

**CONCLUSIONS:**

The MST exhibited adequate validity and excellent reliability and is, therefore, suitable for monitoring the handgrip strength in PD. However, if the goal is to compare the measurements between examiners, the authors recommend that the data be interpreted with caution.

## Introduction

Parkinson's disease (PD) is a chronic disorder of the central nervous system (CNS) characterized by the degeneration of dopaminergic neurons located in the compact part of the substantia nigra, which leads to a decreased production of dopamine, the main neurotransmitter of the nigrostriatal pathway^1^. It is the second most common neurodegenerative disease in individuals >60 years of age, and the prevalence of PD worldwide is estimated to be approximately 100 to 300 cases per 100,000 inhabitants^2^.

The decreased function of the dopaminergic neurons leads to a decrease in spontaneous movements and is responsible for the primary motor symptoms related to PD, including the following: resting tremor, which affects primarily the upper limbs and extends to the neck and face; bradykinesia, characterized by a slowness of voluntary motor activity; muscle stiffness, which results from an inefficient inhibition of the antagonist muscles; postural instability caused by the loss of postural reflexes; and muscle weakness^1^^,^ 3.

The motor sequelae of PD, particularly the gradual loss of muscle strength^4^^-^ 7, cause serious functional limitations and interfere with the performance of activities of daily living (ADLs) and outside tasks. In this sense, the evaluation of muscle strength is essential for the functional evaluation of these individuals and is used in clinical practice for several purposes, including as a functional diagnosis for the assessment of clinical outcomes over time and as a predictive or prognostic indicator^8^ of the occurrence of falls and limitations in ADLs^9^^-^ 12.

Specifically, functional impairment of the upper limbs (ULs) plays an important role in the degree of disability of individuals with PD, and slow muscle contraction and deficits in UL relaxation have been reported^7^. Therefore, the assessment of handgrip strength (HGS) is an important measure because, in addition to evaluating the strength of the upper extremity, HGS has been considered a predictor of overall muscle strength and functional capacity^13^.

In clinical practice, HGS can be evaluated using a portable Jamar dynamometer, which yields objective, valid, accurate, and sensitive HGS measurements^14^. However, the Jamar dynamometer is costly. An alternative method for measuring muscle strength in the clinical setting is the modified sphygmomanometer test (MST) because this test assumes the functions of the portable dynamometer^15^^-^ 17 and is low cost.

The MST involves the use of an aneroid sphygmomanometer, which is a low-cost, portable, readily available device that is commonly acquired by health professionals to measure blood pressure. In addition, the MST can be easily performed by following procedures similar to those adopted in the use of the portable dynamometer and provides objective measurements that can be correlated with the measures of muscle strength^16^^,^ 18 ^,^ 19. Some measurement properties, such as validity and reliability, have been investigated for the MST in some populations with positive results^15^^-^ 24.

However, to date, no studies are available regarding the validity of the MST in PD. Therefore, the present study aimed to assess whether the MST had adequate measurement properties that could be applied to PD patients, thereby providing a new method for evaluating HGS in this population. Specifically, the present study aimed to assess the concurrent criterion validity of the modified sphygmomanometer, to compare the MST with the Jamar dynamometer, and to evaluate the reproducibility (i.e. reliability and agreement) of the MST.

## Method

### Participants

A total of 50 individuals were enrolled in the study. Of these, 24 were recruited from the Brazilian Parkinson Association and formed the group with PD, with mild to moderate motor impairment classified according to the Hoehn and Yahr scale^25^. The control group consisted of 26 healthy older individuals recruited from the Physical Therapy Clinic of the *Universidade Nove de Julho* (UNINOVE) in the state of São Paulo, Brazil.

### Eligibility criteria

For the individuals with PD, the following inclusion criteria were used: preserved cognitive functions assessed with the Mini Mental State Examination; a minimum HGS of 2, based on the assessment by Kendall et al.^26^; the absence of pain in the upper limbs that might have limited the performance of the test; a level ≤3 on the Hoehn and Yahr scale^25^ and being in the "on" period at the time of evaluation. The exclusion criteria included PD patients with deformities or limitations in the range of motion of the wrist and fingers that could prevent the correct use of the measuring devices, having undergone any upper limb surgery in the last 12 months, and the presence of decreased tactile somatosensory sensitivity in the hands and fingers. For the evaluation of sensitivity, a small brush was brushed on the skin. The volunteer subjects closed their eyes during the procedure, and those who did not report tactile sensation were excluded.

The control group, made up of healthy older individuals, was also evaluated with the same inclusion criteria, except for the use of the Hoehn and Yahr scale^25^.

### Ethical aspects

This study followed the principles of the Helsinki Declaration and the Guidelines and Rules for research involving humans that were formulated by the National Health Council of the Ministry of Health and established in Brazil in October 1996.

All of the participants signed an informed consent form and were informed that they could discontinue the study at any stage without penalty. This study was reviewed and approved by the Research Ethics Committee of UNINOVE under protocol no. 477900/11.

## Instruments

### Evaluation of HGS using a Jamar dynamometer

The HGS was measured bilaterally using a Jamar^(r)^ dynamometer (Fabrication Enterprises Inc., Irvington, New York, USA) set at the second handle position^14^^,^ 27. To perform the test, the subject remained in the sitting position in a chair without armrests, with the shoulder in adduction and neutral rotation, the elbow flexed to 90°, the forearm in a neutral position between supination and pronation, and the wrist slightly extended (i.e. between 0° and 30°) and in neutral deviation^14^. Three measurements were recorded for the calculation of the arithmetic mean^14^^,^ 27 ^-^ 29, with a rest period of 20 seconds between each measurement on the same hand^14^^,^ 27. This evaluation procedure is recommended by the American Society of Hand Therapists^27^ and has been reproduced in studies using Brazilian subjects^28^^,^ 29.

After a 3-minute interval, the same procedure was repeated on the other hand, restarting the test using the next device. The order of application of the instruments was determined by drawing by lot performed by the subjects.

### Evaluation of HGS using the modified sphygmomanometer test

The modifications made to the sphygmomanometer were based on previously described methods^17^^,^ 18 ^,^ 30 ^,^ 31 and were adapted according to the dimensions and shape of the Jamar dynamometer. For this purpose, the dimensions of the Jamar dynamometer were measured with the handle set at the second position, and a metal bar with the same size (10x5x2 cm) was covered with a paste made of cornstarch and white glue. When dry, this paste became solid and did not deform under handgrip pressure.

**Figure 1.**
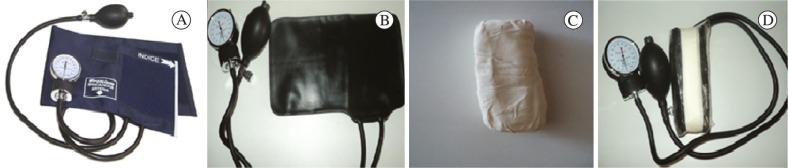
(A) sphygmomanometer in the original format; (B) inner sphygmomanometer cuff; (C) modified device (10X5X2 cm); (D) modified sphygmomanometer.

For the sphygmomemnometer test instrument, a Premium brand (Fabrication Accumed LTDA., Rio de Janeiro, Rio de Janeiro, Brazil) aneroid sphygmomanometer was used. The modification involved the removal of the outer cloth cuff and Velcro from the device; only the inner cuff (i.e. the bladder) was used because, according to Souza et al.^32^, the inner cuff could be more easily adapted for training and exhibited better stabilization compared to other adaptations. The device made with the metal bar and paste was wrapped with the cuff and fixed longitudinally with adhesive tape. The device was then sealed with clear polyvinyl chloride (PVC) film and secured with tape (Figure 1).

The MST was performed with the sphygmomanometer pre-inflated to 80 mm Hg^17^; the subject remained in the sitting position in a chair without an armrest, with the shoulder in adduction and neutral rotation, the elbow flexed to 90°, and the forearm in neutral rotation, and the wrist in a neutral deviation and slightly extended (between 0° and 30°), and then, at a simple and precise command of the examiner, the subject performed the handgrip test. The subject was asked to hold each contraction for 5 seconds, and then a rest period of 20 seconds was allowed between measurements of the same arm^14^^,^ 27. The MST was performed bilaterally four times, with the first measurement being performed to familiarize the subject with the device. The arithmetic mean of the last three measurements was used as the study outcome^14^^,^ 27 ^-^ 29.

To obtain an exact measurement, the sphygmomanometer was calibrated periodically (once a year). Considering the need to safeguard the health of the patient and ensure reliable measurements, we followed the Metrological Technical Regulation, which establishes the conditions that the mechanical aneroid sphygmomanometers should meet. According to this regulation, users must submit their devices yearly to metrological control executed by specialized professionals.

### Validation of the concurrent criterion

The concurrent criterion validity is evaluated when the measure to be validated and the criterion measure are obtained at the same time^33^^,^ 34. Therefore, to assess the concurrent criterion validity, the HGS was obtained for both the Jamar portable dynamometer and modified sphygmomanometer.

### Assessment of reproducibility

To evaluate the interrater reproducibility, two examiners performed the MST independently to prevent the exchange of information.

To evaluate the intra-rater reproducibility, one of the examiners performed the MST on two different occasions, with a maximum period of 7 days between each test. The order of application of the instruments in the second evaluation was the same as that adopted in the first evaluation.

In the period between the tests, those individuals who reported information that could change the HGS test results, such as injuries or pain in the upper limbs, were automatically excluded from the study to avoid interference with the measurement of reproducibility. Patients with PD who were not medicated (in the "on" period) were also excluded.

### Statistical analysis

For the sample characterization and distribution of the measurements obtained, descriptive statistics were performed using the means and standard deviations for the quantitative variables and frequencies for the categorical variables. To compare the HGS values between the control and PD groups, an unpaired Student's *t* test was used.

To analyze the concurrent criterion validity, the correlation between the MST and the portable Jamar dynamometer was assessed. For this purpose, the Pearson correlation coefficient (*r*) was used, considering the strength or magnitude of the correlation between variables, based on the following criteria: weak (correlation coefficient between 0.1 and 0.3), moderate (a value between 0.4 and 0.6), and strong (a value between 0.7 and 0.9)^35^. In addition, a simple linear regression was used as a measure of validity. For this purpose, HGS evaluated with the MST was considered the independent variable, whereas HGS evaluated with the portable dynamometer was considered the dependent variable. It was thus possible to formulate a mathematical equation to predict HGS.

To analyze the reproducibility of the MST, the reliability and the agreement between measures were evaluated at three different periods. To assess reliability, the intraclass correlation coefficient (ICC, type 2.1)^36^ and the respective 95% confidence interval (CI) for the ICC were used (ICC of 0.80-0.99 = excellent; ICC of 0.60-0.79 = good, and ICC <0.60 = weak)^37^. To analyze the intra- and interrater agreement, two measures were used-the Standard Error of Measurement (SEM) and the Minimum Detectable Change (MDC)^38^. The SEM reflects the instrument error and was calculated by dividing the standard deviation (SD) of the mean difference by the square root of 2 (SD of the differences/√2). The MDC is the minimum change of the measurement that can be interpreted as real change and was calculated using the formula MDC = 1.96 x√2 x SEM 38.

The interrater agreement was measured using the Bland-Altman plot. Using this test, scatter plots were constructed, which revealed the individual differences (y-axis) according to the means observed in both evaluations (x-axis)^39^.

The Bland-Altman plots were made using the MedCalc statistical software, whereas the remaining analyses were performed using SPSS for Windows (SPSS. Inc., Chicago, IL, USA). For all the analyses, a risk of α≤0.05 was considered significant.

## Results

A total of 36 subjects with PD were recruited, but 9 of these were excluded because of positive cutoff values during the screening for a cognitive deficit, and 3 had pain in the upper limbs; therefore, the sample consisted of 24 individuals with PD. For the control group, 27 healthy subjects were recruited, and, of these, only 1 was excluded for having had orthopedic surgery in the right upper limb within the last 12 months; therefore, 26 older subjects formed the control group.

The final sample consisted of 50 subjects, whose clinical and demographic characteristics are presented in Table 1. In addition, no significant difference (p>0.05) was observed in the assessment of the HGS between the control and PD groups, demonstrating that the groups were homogeneous.

**Table 1.** Demographic and clinical characteristics of the study subjects.

Variable	CG elderly (n=26)	Parkinsonian (n=24)
Men	11 (42%)	10 (42%)
Women	15 (58%)	14 (58%)
Age (years)	63.4 (7.2)	65.5 (6.2)
BMI (kg/m2)	22.5 (3.9)	24.9 (2.0)
Right UL dominant	19 (73%)	18 (75%)
Left UL dominant	7 (27%)	6 (25%)
Hoehn and Yahr Classification	- -	2 (1/3)
Time since PD diagnosed (years)	- -	7.2 (4.1)
MST Right (mm Hg)		
First evaluation	55.95 (21.28)	59.35 (20.34)
Second evaluation	56.25 (21.02)	67.43 (21.25)
Third evaluation	60.22 (20.43)	65.71 (14.64)
MST Left (mm Hg)		
First evaluation	57.15 (16.73)	60.66 (18.35)
Second evaluation	55.38 (15.97)	70.91 (23.21)
Third evaluation	66.15 (25.24)	72.96 (17.66)

CG: control group; PD: Parkinson's disease; BMI: body mass index; UL: upper limb; MST: modified sphygmomanometer test. The data are expressed as the frequency (percentage), median (interquartile range), or mean (standard deviation).

A moderate correlation was observed between the measurements obtained with the MST and the Jamar dynamometer in the groups evaluated (Table 2). The simple linear regression test indicated moderate predictive values, except for the HGS in the left arm of subjects with PD, whose predictive values were low. Table 2 shows the regression equation that predicted HGS.

**Table 2.** Pearson correlation coefficient and simple linear regression analysis between the modified sphygmomanometer test and the portable Jamar dynamometer.

	Correlation coefficient (*r*)	Simple linear regression (*r* ^2^)	Regression Equation
HGS R (CG)	0.67*	0.41	y=1.4562 + 2.1532x
HGS L (CG)	0.65*	0.43	y=8.6356 + 1.9897x
HGS R (PD)	0.68*	0.46	y=0.9996 + 1.7848x
HGS L (PD)	0.45*	0.20	y=20.7755 + 1.3719x

CG: control group; PD: Parkinson's disease; HGS: handgrip strength; R: right; L: left.

* (P<0.05).

With regard to reproducibility, adequate, good, and excellent degrees of reliability were observed in both groups (Table 3). For agreement, the SEM varied between 2.29 in the control group and 2.67 in the PD group, whereas MDC varied between 6.34 in the control group and 7.40 in the PD group. Table 3 indicates that the values in both groups were similar.

**Table 3.** Reproducibility (reliability and agreement) of the modified sphygmomanometer test (MST).

	Reliability ICC_2,1_ (IC 95%)	Agreement SEM	Agreement MDC
MST R (CG)	0.79 (0.55-0.95)	2.56	7.09
MST L (CG)	0.88 (0.75-0.95)	2.29	6.34
MST R (PD)	0.89 (0.62-0.96)	2.55	7.06
MST L (PD)	0.83 (0.50-0.95)	2.67	7.40

MST: modified sphygmomanometer test; R: right; L: left. CG: control group; PD: Parkinson's disease; ICC: intraclass correlation coefficient; SEM: standard error of measurement; MDC: minimum detectable change.

**Figure 2.**
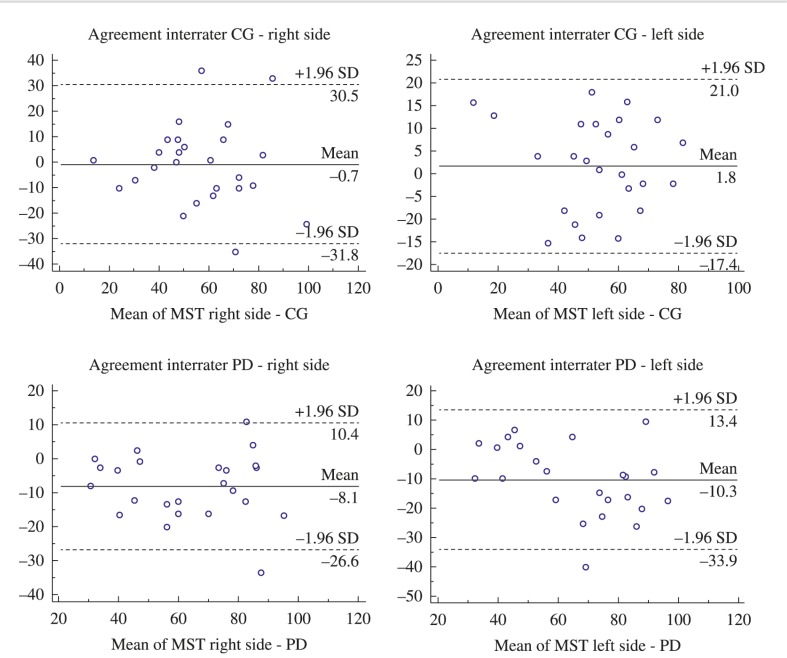
Interrater agreement according to the Bland-Altman method. CG: control group; PD: Parkinson's disease; MST: Modified sphygmomanometer test; SD: standard deviation.

Figure 2 illustrates the interrater agreement in both groups. When the mean difference of the measurements obtained by different examiners was compared, a symmetrical distribution was observed around the mean. However, wide limits of agreement and a high bias were observed, particularly in the PD group.

## Discussion

The HGS is often affected in subjects with PD because of motor changes during disease progression, and these changes negatively impact the performance of ADLs and self-care. Considering the chronic degeneration that occurs in PD patients, HGS must be constantly monitored by therapists using reliable and easily accessible devices.

Accordingly, the purpose of this study was to analyze the concurrent criterion validity and reproducibility of the MST for the assessment of HGS in individuals with PD. The results indicate a moderate correlation between the MST and the portable dynamometer, and the reproducibility of the MST was considered adequate, good, or excellent.

The concurrent criterion validity indicates the adequacy of the instrument using distinct data, including those obtained from the gold-standard measurements. In this sense, a positive and moderate correlation was observed between the MST and the Jamar dynamometer, which is considered the gold standard for evaluating HGS^27^. Furthermore, the MST values could moderately predict the HGS assessed with the Jamar dynamometer, except on the left side, which exhibited a low predictive value. Therefore, it can be inferred that the measurements assessed by both instruments were similar.

Additionally, the present study aimed to evaluate the reproducibility of the MST measurements, defined as the ability of an instrument to yield reliable results even when used by different examiners or during different periods^40^. The reliability of the MST has been tested on different populations (adults and healthy older individuals with rheumatoid arthritis and lower back pain), and the measurements obtained were adequate^15^^-^ 24. However, to date, the reliability of the MST had not been tested in individuals with PD^41^.

Reproducibility studies (i.e. reliability and agreement) are crucial in assessing the variability of a method or instrument and, consequently, in avoiding the misinterpretation of variables before and after interventions. Regarding reliability, adequate, good, and excellent intra- and interrater ICC values were observed in both groups. Therefore, the MST is a valid and reliable method for measuring HGS in individuals with PD.

With regard to the intra- and interrater agreement assessed using the SEM and MDC^42^, a small SEM was obtained, and therefore, it is expected that the measurements made in the same individual at different times would have a variation of 2.67 mmHg, which is related to the measurement error and not to changes in the clinical status of the patient. The MDC values found indicate that a change >7.40 mmHg has a <5% probability of occurring due to random variation or a random error in the measurement.

Of note, the mean difference between the control and PD groups on the left side exceeded the values established by the SEM and MDC. This variation may be attributed to the non-dominance of the left hand^43^, considering that most subjects were right-handed.

Although the interrater agreement was assessed using the Bland-Altman plot, no satisfactory results were obtained. The plots showed a high bias and wide limits of agreement, particularly in the PD group. The Bland-Altman plot has been used in various reliability studies^44^. However, it was not possible to compare the results obtained herein with those of other studies because no previous studies used this method to analyze the reliability of the MST in this particular population.

One of the limitations of this study was related to the use of a sample composed of individuals with mild to moderate PD. In this respect, previous studies have shown that individuals with more severe signs and symptoms of PD tend to have cognitive deficits that interfere with or even prevent the adequate performance of the HGS test^45^. Therefore, in this study, individuals classified as levels 4 and 5 in the Hoehn and Yahr scale were excluded. However, further studies should be conducted to verify whether the results presented herein are observed in subjects with more severe impairments and whether the severity of motor symptoms and postural changes, which are frequent in patients in the advanced stages of PD, interfere with the performance of this analysis.

In summary, it can be concluded that, despite the above limitations, the results reported herein are relevant to the field of physical therapy and the rehabilitation of patients with PD because the results corroborate the adequate validity and reliability of the MST. However, if the goal is to compare the measurements made by distinct examiners, the data should be interpreted with caution. Therefore, HGS, which is considered a predictor of overall muscle strength^13^, can be assessed more adequately in the future. By doing so, the planning of treatment strategies and the progression of PD can be monitored more adequately by the therapist and at a low cost.

## References

[1] Wirdefeldt K, Adami HO, Cole P, Trichopoulos D, Mandel J (2011). Epidemiology and etiology of Parkinson's disease: a
review of the evidence. Eur J Epidemiol.

[2] de Lau LM, Breteler MM (2006). Epidemiology of Parkinson's disease. Lancet Neurol.

[3] Jankovic J (2008). Parkinson's disease: clinical features and
diagnosis. J Neurol Neurosurg Psychiatry.

[4] Cano-de-la-Cuerda R, Pérez-de-Heredia M, Miangolarra-Page JC, Muñoz-Hellín E, Fernández-de-Las-Peñas C (2010). Is there muscular weakness in Parkinson's
disease?. Am J Phys Med Rehabil.

[5] Friedman JH, Abrantes AM (2012). Self perceived weakness in Parkinson's
disease. Parkinsonism Relat Disord.

[6] Moreno Catalá M, Woitalla D, Arampatzis A (2013). Central factors explain muscle weakness in young fallers
with Parkinson's disease. Neurorehabil Neural Repair.

[7] Neely KA, Planetta PJ, Prodoehl J, Corcos DM, Comella CL, Goetz CG (2013). Force control deficits in individuals with Parkinson's
disease, multiple systems atrophy, and progressive supranuclear
palsy. PLoS ONE.

[8] Attivissimo F, Cavone G, Lanzolla AML, Savino M (2009). Application of hand grip signals for an objective
evaluation of Parkinson disease: Analysis and comparison with standard functional
clinical tests. Measurement.

[9] Gazibara T, Pekmezovic T, Tepavcevic DK, Tomic A, Stankovic I, Kostic VS (2014). Circumstances of falls and fall-related injuries among
patients with Parkinson's disease in an outpatient setting. Geriatr Nurs.

[10] Mak MK, Pang MY, Mok V (2012). Gait difficulty, postural instability, and muscle
weakness are associated with fear of falling in people with Parkinson's
disease. Parkinsons Dis.

[11] Rudzińska M, Bukowczan S, Stożek J, Zajdel K, Mirek E, Chwała W (2013). Causes and consequences of falls in Parkinson disease
patients in a prospective study. Neurol Neurochir Pol.

[12] Mak MK, Pang MY (2010). Parkinsonian single fallers versus recurrent fallers:
different fall characteristics and clinical features. J Neurol.

[13] Curb JD, Ceria-Ulep CD, Rodriguez BL, Grove J, Guralnik J, Willcox BJ (2006). Performance-based measures of physical function for
high-function populations. J Am Geriatr Soc.

[14] Figueiredo I, Sampaio RF, Mancini MC, Silva FCM, Souza MAP (2007). Teste de força de preensão utilizando o dinamômetro
Jamar. Acta Fisiátrica.

[15] Helewa A, Goldsmith CH, Smythe HA (1986). Patient, observer and instrument variation in the
measurement of strength of shoulder abductor muscles in patients with rheumatoid
arthritis using a modified sphygmomanometer. J Rheumatol.

[16] Helewa A, Goldsmith CH, Smythe HA (1993). Measuring abdominal muscle weakness in patients with low
back pain and matched controls: a comparison of 3 devices. J Rheumatol.

[17] Helewa A, Goldsmith CH, Smythe HA (1981). The modified sphygmomanometer-an instrument to measure
muscle strength: a validation study. J Chronic Dis.

[18] Lucareli PRG, Lima MO, Lima FPS, Gimenes RO, Lucareli JGA, Junior SAG (2010). Comparação dos métodos de mensuração da força muscular
dos flexores dos dedos das mãos através da dinamometria manual e esfigmomanômetro
modificado. Rev Einstein.

[19] Rice CL, Cunningham DA, Paterson DH, Rechnitzer PA (1989). Strength in an elderly population. Arch Phys Med Rehabil.

[20] Bohannon RW, Lusardi MM (1991). Modified sphygmomanometer versus strain gauge hand-held
dynamometer. Arch Phys Med Rehabil.

[21] Balogun JA, Akomolafe CT, Amusa LO (1990). Reproducibility and criterion-related validity of the
modified sphygmomanometer for isometric testing of grip strength. Physiother Can.

[22] Hamilton GF, McDonald C, Chenier TC (1992). Measurement of grip strength: validity and reliability
of the sphygmomanometer and jamar grip dynamometer. J Orthop Sports Phys Ther.

[23] Perossa DR, Dziak M, Vernon HT, Hayashita K (1998). The intra-examiner reliability of manual muscle testing
of the hip and shoulder with a modified sphygmomanometer: a preliminary study of
normal subjects. J Can Chiropr Assoc.

[24] Isherwood L, Lew L, Dean E (1989). Indirect evidence for eccentric muscle contraction
during isometric muscle testing performed with a modified
sphygmomanometer. Physiother Can.

[25] Goetz CG, Poewe W, Rascol O, Sampaio C, Stebbins GT, Counsell C (2004). Movement Disorder Society Task Force report on the Hoehn
and Yahr staging scale: status and recommendations. Mov Disord.

[26] Kendall FP, McCreary EK, Provance PG (2007). Músculos, provas e funções.

[27] Fess EE (1992). Grip strength.

[28] Reis MM, Arantes PMM (2011). Medida da força de preensão manual- validade e
confiabilidade do dinamômetro saehan. Fisioter Pesqui.

[29] Geraldes AAR, Oliveira ARM, Albuquerque RB, Carvalho JM, Farinatti PTV (2008). A força de preensão manual é boa preditora do desempenho
funcional de idosos frágeis: um estudo correlacional múltiplo. Rev Bras Med Esporte.

[30] Delgado C, Fernandes JF, Barbosa FP, Oliveira HB (2004). Utilização do esfigmomanômetro na avaliação da força dos
músculos extensores e flexores da articulação do joelho em
militares. Rev Bras Med Esporte.

[31] Kaegi C, Thibault MC, Giroux F, Bourbonnais D (1998). The interrater reliability of force measurements using a
modified sphygmomanometer in elderly subjects. Phys Ther.

[32] Souza LAC, Martins JC, Moura JB, Teixeira-Salmela LF, De Paula FVR, Faria CDCM (2014). Assessment of muscular strength with the modified
sphygmomanometer test: what is the best method and source of outcome
values?. Braz J Phys Ther.

[33] Portney LG, Watkins MP (2000). Foundations of clinical research: applications to
practice.

[34] Sim J, Arnell P (1993). Measurement validity in physical therapy
research. Phys Ther.

[35] Dancey CP, Reidy J (2006). Estatística sem matemática para psicologia: usando SPSS para
Windows.

[36] Krebs DE (1986). Declare your ICC type. Phys Ther.

[37] McGraw KO, Wong SP (1996). Forming inferences about some intraclass correlation
coefficiens. Psychol Methods.

[38] Terwee CB, Bot SD, de Boer MR, van der Windt DA, Knol DL, Dekker J (2007). Quality criteria were proposed for measurement
properties of health status questionnaires. J Clin Epidemiol.

[39] Bland JM, Altman DG (1986). Statistical methods for assessing agreement between two
methods of clinical measurement. Lancet.

[40] de Vet HC, Terwee CB, Knol DL, Bouter LM (2006). When to use agreement versus reliability
measures. J Clin Epidemiol.

[41] Souza LAC, Martins JC, Teixeira-Salmela LF, Godoy MR, Aguiar LT, Faria CDCM (2013). Avaliação da força muscular pelo teste do
esfigmomanômetro modificado: uma revisão da literatura. Fisioter Mov.

[42] Magalhães MO, Costa LOP, Ferreira ML, Machado LAC (2011). Clinimetric testing of two instruments that measure
attitudes and beliefs of health care providers about chronic low back
pain. Rev Bras Fisioter.

[43] Incel NA, Ceceli E, Durukan PB, Erdem HR, Yorgancioglu ZR (2002). Grip strength: effect of hand dominance. Singapore Med J.

[44] Andrade JA, Figueiredo LC, Santos TRT, Paula AC, Bittencourt NF, Fonseca ST (2012). Reliability of transverse plane pelvic alignment
measurement during the bridge test with unilateral knee extension. Rev Bras Fisioter.

[45] Zhu K, van Hilten JJ, Marinus J (2014). Predictors of dementia in Parkinson's disease; findings
from a 5-year prospective study using the SCOPA-COG. Parkinsonism Relat Disord.

